# Atrophiæ Sulphas in Acute Myringitis and Otitis

**Published:** 1877-02

**Authors:** A. N. Ellis

**Affiliations:** Joliet, Illinois


### ATROPIÆ SULPHAS IN ACUTE MYRINGITIS AND OTITIS.

By A. N. ELLIS, M.D., Joliet, Illinois.

No one who has ever treated a case of acute inflammation of the ear need be reminded of the terrible sufferings of his patient, the importance of prompt action, as well as the limited means at his command of giving instant relief.

From the situation of the drum, as well as its peculiar anatomical construction, it is very liable to become affected.

Myringitis, as its name indicates, is an acute inflammation of the membrana tympani. It is not a very common disease, yet physicians meet with it occasionally. It is not confined to any class — all may have it, from the robust to the weakest. Traumatic myringitis is the result of some physical injury. I will refer to two cases of the kind before closing this paper.

The exciting causes of idiopathic myringitis are many. Exposure in damp inclement weather, strong cold winds, bathing in cold water, passing suddenly from the warm and close air of a crowded hall to the street, sleeping in a bed over which passes a current of cold air, etc., etc. Often no immediate cause can be discovered.

Acute myringitis often comes on very suddenly without any premonition whatever; suddenly the patient is aroused from sleep. And how he suffers! Were you to pass a dagger through him he could not suffer more acutely. The pain is almost unendurable. I have seen several cases where the patient became almost frantic. The pain soon extends to the surrounding parts — to the head, face, teeth, jaws, and more or less down the neck. The movements of the jaw in talking or chewing increase it. The auricle soon becomes affected; is exceedingly tender to the touch. The hearing is dull, often lost; there is a sense of fullness and pressure. *Tinnitus aurium* supervenes, and is very annoying. *Autophonia* is a very troublesome subjective symptom.

*Objective symptoms.*—The objective symptoms, though not many, are very characteristic. The trouble is confined to the drum. A congestion of the membrane is observable. The vessels along the handle of the malleus are injected; the individual vessels are lost in the general redness; the cone of light is obscured. The surface of the membrane may appear convex externally, and has a dark, red, smooth flesh color. The line of union between the skin and membrane is entirely obliterated. After the first few days of agonizing pain and annoying *tinnitus aurium*, an abscess often forms in the membrana tympani, and perforation takes place, followed at once by suppuration. Untreated, the otorrhœa is followed by a long train of evils.

Take in the anatomy of the region at a glance, and what do we find? The lining of the external auditory canal functionates anteriorly as common integument, posteriorly as mucous membrane. The roof of the cavity is thin. Examine it in the skeleton, and you will find it so fragile that it is almost translucent. Lying immediately upon it are the membranes of the brain. Under the floor of the cavity is the jugular vein; anteriorly is found the largest artery of the head — the internal carotid — with the venous sinus in close proximity. These are separated from the cavity of the middle ear by very delicate lamellæ. The inner or labyrinth wall offers but slight resistance to the transition of the inflammation to the facial nerve, fenestrae and internal ear. Who will say that an unchecked inflammation and discharge in this important region is not a dangerous thing? And yet who has not heard physicians give their patients such advice as this: "Let it alone; you will outgrow it. It is dangerous to check a flow from the ear." Can anything be more unreasonable? Is disease safer than health? Otorrhœa *does not get well* as a rule without treatment!

Untreated, the disease soon extends to the neighboring parts, often resulting in otitis externa and interna; fungus growths springing from the cavity of the middle ear; anchylosis of the stapes; closure of the eustachian tube; deafness; caries of the temporal bone, cerebral meningitis! Only the unfortunate who have suffered from these things can fully realize their dire results. Only he who has day after day, week after week, and month after month battled with these troublesome afflictions can estimate the amount of time, patience and skill necessary to bring them to a favorable termination.

Having often witnessed the happy effects of the active principle of belladonna in painful affections of the eye, I was led to use it in the treatment of the ear where an anodyne was demanded.

About six years ago I was called to see a man employed at the building of the iron railroad bridge across the Missouri at Fort Leavenworth. He complained of great pain in his left ear. It had come on suddenly while he was laboring in one of those iron caissons where the pressure of the air was great. The diagnosis was easy, showing a case of traumatic myringitis. Placing the patient in bed, a few drops of the atropine solution (atropiæ sulph. grs. iv., ad aquæ ℥ i,) were instilled into the ear. Six leeches were applied to the mastoid process, six more in front of the meatus externus. He was discharged on the second day cured.

About four years ago, at Fort Craig, New Mexico, a soldier while standing near a cannon which was being fired was taken with great pain in his right ear. I was absent from the post at the time, and did not see him for some hours. He was lying in the hospital, and complained of great suffering. Blood was seen issuing from the affected ear. Carefully syringing the canal, illumination showed fracture of the malleus. Acute myringitis supervened at once. He was treated with the atropine solution. The artificial leech was used both before and immediately behind the meatus extermus, and about four ounces of blood taken. The results were all that could be desired.

Since that time I have used the atropia in about one dozen cases, and with gratifying success. The medicine should always be warmed slightly by dipping the bottle in warm water before using. Conjoined with the use of leeches, the recumbent position, perfect rest, and the careful exclusion of all exciting causes, I have not seen a case where the remedy failed.

---

Chicago and other American Cities would do well to establish a system of hospital telegraphy similar to that which has recently proved useful in Paris. All the hospitals and dispensaries are placed in telegraphic communication with a central bureau, where admissions to each institution are obtainable. Thus, without delay, without the often painful and sometimes dangerous journeyings hither and thither, of patients in search of shelter and treatment, information can speedily be obtained upon all requisite points.

Chicago, in particular, separated as its three grand divisions are, would offer great facilities of this character, if a bureau of hospital telegraphy were located in its business center.

